# Evaluation of the biodistribution of [^99m^Tc]Tc-PSMA-GCK01 SPECT/CT in a prospective, intraindividual comparison with [^68^Ga]PSMA-11 PET/CT for phenotyping in mCRPC prior to PSMA-targeted radioligand therapy

**DOI:** 10.1186/s13550-026-01501-0

**Published:** 2026-08-18

**Authors:** Emil Novruzov, Eduards Mamlins, Joseph Kabunda, Jens Cardinale, Milani Qebetu, Clemens Kratochwil, Anna Feisthauer, Stefan Martlbauer, Chimbabantu Alexis Kaoma, Sandile Sibiya, Tertia de Bruin, Sipho Mdanda, Mbongeni Shungube, Cindy Davis, Liani Smith, Yonwaba Mzizi, Kgomotso Mokoala, Honest Ndlovu, Keamogetswe Ramonaheng, Frederik L. Giesel, Mike M Sathekge

**Affiliations:** 1 Department of Nuclear Medicine, Medical Faculty and University Hospital Duesseldorf, Heinrich-Heine-University Duesseldorf, Duesseldorf, Germany; 2 Department of Nuclear Medicine, University of Pretoria and Steve Biko Academic Hospital, Pretoria, South Africa; 3 Nuclear Medicine Research Infrastructure (NuMeRI), Pretoria, South Africa; 4 Department of Nuclear Medicine, University Hospital Heidelberg, Heidelberg, Germany; 5 Centre for Integrated Oncology (CIO) Duesseldorf, CIO Aachen-Bonn-Cologne-Duesseldorf, 40225 Duesseldorf, Germany

**Keywords:** PSMA SPECT, ^99m^Tc, PSMA-GCK01, phenotyping, PSMA radioligand therapy, biodistribution

## Abstract

**Background:**

PSMA-PET/CT became an important means for prostate cancer management. The high-resolution and signal-to-noise ratio of PET is mandatory in primary staging or biochemical relapse that often present with tiny lesions. PSMA-PET is also used to select patients for PSMA-targeted radioligand-therapy, but scanner availability increasingly becomes a bottleneck. The resolution of PSMA-SPECT/CT might be sufficient to tailor PSMA positive vs. negative disease and would help overcome supply limitations. This prospective, monocentric study evaluates the biodistribution and tumor uptake of [^99m^Tc]Tc-PSMA-GCK01 in an intra-individual head-to-head comparison with [^68^Ga]PSMA-11 to assess its suitability for phenotyping prior to RLT.

**Results:**

A cohort of ten patients (median age, 68 y; range, 56–85 y) with advanced-stage, metastatic prostate cancer underwent both unenhanced [^68^Ga]PSMA-11 PET/CT and [^99m^Tc]Tc-PSMA-GCK01 SPECT/CT scans with a mean time interval of 6 days (± 1). The tracer uptake in malignant lesions and normal organs was assessed visually and semi-quantitatively using spherical VOIs by two nuclear medicine physicians in consensus. Compared to [^99m^Tc]Tc-PSMA-GCK01, [^68^Ga]PSMA-11 displayed a trend toward higher uptake in normal organs. Renal tracer uptake was significantly higher with [^68^Ga]PSMA-11 than with [^99m^Tc]Tc-PSMA-GCK01. In the salivary glands and liver, there was no statistically significant difference in uptake between the two tracers, although [^99m^Tc]Tc-PSMA-GCK01 displayed a trend toward lower uptake.

**Conclusion:**

Our study demonstrates intraindividual comparability of [^99m^Tc]Tc-PSMA-GCK01 and [^68^Ga]PSMA-11 with respect to normal-organ distribution including typical reference organs such as the liver or salivary glands. If only lesions > 2 cm are considered, [^99m^Tc]Tc-PSMA-GCK01 PSMA-SPECT seems to be a viable, cost-effective alternative to [^68^Ga]PSMA-11 to stratify patients toward or against PSMA-RLT in certain clinical settings.

**Supplementary Information:**

The online version contains supplementary material available at 10.1186/s13550-026-01501-0.

## Introduction

The original approval of [^68^Ga]PSMA-11 has transformed prostate cancer clinical practice and supported advancement of PSMA-targeting theranostics. The VISION trial used lesion uptake above liver activity (visual assessment; i.e., phenotyping) on [^68^Ga]PSMA-11 imaging as a key prerequisite for RLT eligibility and excluded patients with measurable lesions showing uptake equal to or lower than liver; measurable disease was defined on anatomic imaging (e.g., lymph nodes > 2.5 cm short axis, solid-organ metastases > 1.0 cm short axis, and bone metastases with a soft-tissue component > 1.0 cm short axis). With the globally high incidence and prevalence of prostate cancer, demand for PSMA PET continues to increase. However, in real-life settings, patients in remote or resource-limited regions may not always have sufficient access to PSMA-PET infrastructure [1, 2].

Single-photon emission computed tomography with unenhanced low-dose computed tomography (SPECT/CT) systems are widely available globally and may help expand access to PSMA theranostics to patients in regions where PSMA PET is not available. Additionally, ^99^Mo/^99m^Tc generators enable decentralized and cost-effective production of radionuclides, supporting large-scale clinical use compared with ^68^Ga generators [3–6]. Wang et al. reported a meta-analysis suggesting the potential of ^99m^Tc-labelled PSMA ligands to provide supplementary information to [^68^Ga]PSMA-11 PET, supporting the utility in resource-limited settings [7].

Recently, Cardinale et al. introduced a novel ^99m^Tc/^188^Re theranostic tandem targeting PSMA, i.e. [^99m^Tc]Tc/[^188^Re]Re-PSMA-GCK01, which aimed to have a similar biodistribution as currently available small-molecule-based RLT and, for the first time, provide an alternative theranostic platform with the employment of generator-based (^188^W/^188^Re generator) ^188^Re-labelling. This concept sought to decentralize prostate cancer management and, thus, overcome potential isotope supply limitations resulting from lack of high-flux neutron facilities and PET infrastructure, remote areas with restricted airfreight frequency, and resource-limited healthcare systems. Previous results supported the comparability of [^99m^Tc]Tc-PSMA-GCK01 to other existing ^99m^Tc-labelled PSMA tracers in terms of biodistribution and tumor uptake [8–10]. We consider the phenotyping prior to RLT as the most promising application area of ^99m^Tc-labelled PSMA-ligands that warrants validation within regular clinical care.

This prospective, monocentric study sought to evaluate the biodistribution and tumor uptake of [^99m^Tc]Tc-PSMA-GCK01 compared with [^68^Ga]PSMA-11 to assess its eligibility for phenotyping prior to RLT.

## Materials and methods

### Patient cohort

A cohort of ten patients (median age, 68 y; range, 56–85 y) with multi-organ metastasized, advanced - stage prostate cancer underwent both unenhanced [^68^Ga]PSMA-11 PET/CT and [^99m^Tc]Tc-PSMA-GCK01 SPECT/CT scans with a mean time interval of 6 days (± 1). This prospective monocentric study was approved by the Institutional Ethics Committee at the University of Pretoria, South Africa. Written informed consent was obtained from all patients. In brief, the inclusion criteria for this study were informed consent by patients and an indication for RLT based on previous therapy course. Patient data were pseudonymized before analysis.

### Imaging procedures and radiopharmaceuticals

Unenhanced [^68^Ga]PSMA-11 PET/CT and [^99m^Tc]Tc-PSMA-GCK01 SPECT/CT scans were acquired on different days with a mean time interval of 6 days (± 1) to avoid potential carryover effects or tracer interference. All unenhanced total-body PET/CT scans were acquired on a Biograph mCT 40 PET/CT scanner (Siemens). Accordingly, total-body SPECT/CT scans were acquired on a double-head hybrid gamma camera (Symbia, Siemens Healthineers). *Supplemental Table 1* summarizes the acquisition protocols for both scans.

[^99m^Tc]Tc-PSMA-GCK01 was synthesized as described previously [8]. There was no special preparation before the scans and the patients were observed for at least 30 min following the SPECT/CT scan. SPECT/CT was performed on average 120 (± 15) minutes following the injection of [^99m^Tc]Tc-PSMA-GCK01 with a mean injected dose of 500 (± 30) MBq. PET/CT was acquired on average 90 (± 5) minutes following the injection of [^68^Ga]PSMA-11 with mean injected dose of 300 (± 20) MBq.

### Image analysis

The tracer uptake in malignant lesions and normal organs was assessed visually and semi-quantitatively by using spherical VOIs by two experienced nuclear medicine physicians in consensus using the dedicated reading software program Hermia Medical Imaging (Suite v6.1, Affinity 5.01, Hermia Medical Solutions AB, Strandbergsgatan 16, 11251 Stockholm, Sweden). The data required for the analysis were prepared and pre-configured by a well-trained scientific staff member (AF). For each metastatic site (skeletal system, lymph nodes and visceral), up to three representative lesions per patient were selected for further analysis. For both imaging modalities, the mediastinal blood pool in the descending aorta and pelvic fat tissue (on the patient’s right side) were used as background to ensure a consistent comparison of biodistribution between the tracers. Because PET SUVs and SPECT counts are not directly comparable as absolute measures, uptake was expressed as dimensionless ratios, including target-to-background (TBR) and target-to-blood pool (TBP) ratios, calculated within each modality. Additionally, normal tissues (salivary and lacrimal glands, pancreas, and kidneys) were evaluated with a 2-cm-diameter spherical VOI, whereas bladder, brain, lung, liver, and spleen were evaluated with a 4-cm-diameter spherical VOI placed within the organ parenchyma. Metastases, blood pool and background were assessed with a 2-cm-diameter spherical VOI. Given that patients with advanced mCRPC frequently undergo multiple imaging scans across both molecular and conventional modalities, we utilized a composite reference method as the validation standard for lesion evaluation.

### Statistical analysis

We used descriptive analyses for tumor characteristics and tracer uptake. Following standardization of tracer uptake metrics for both scans using TBP and TBR-normalized tracer uptake metrics, tumor and normal tissues were analyzed via paired t tests or Wilcoxon signed rank tests. A p value of < 0.05 was considered to indicate statistical significance. All the statistical analyses were performed via SigmaStat Version 3.5 (Systat Software, Inc., San Jose, CA, USA) and SigmaPlot Version 11.0 (Systat Software, Inc., San Jose, CA, USA) for graphical visualization.

## Results

A cohort of 10 biologically male patients (median age, 68 y; range, 56–85 y) was included in this study. Table 1 depicts the patient characteristics. No immediate adverse events were observed during the post-scan observation period following [^99m^Tc]Tc-PSMA-GCK01 administration. Visual assessment by the readers revealed qualitative concordant lesion detection between the two tracers in this high-disease burden cohort.

**Table 1:** Overview of patient characteristics and imaging parameters

Parameters	Mean ± SD
Age (years) Median; range	68 (56–85)
PSA (ng/mL)	276.1 (± 613)
Injected activity (MBq)	
[^99m^Tc]Tc-PSMA-GCK01	500 (± 30)
[^68^Ga]PSMA-11	300 (± 20)
Acquisition Time after Radiotracer Administration (min)	
[^99m^Tc]Tc-PSMA-GCK01	120 (± 15)
[^68^Ga]PSMA-11	90 (± 5)

### Biodistribution and tumor uptake

**Fig. 1:**
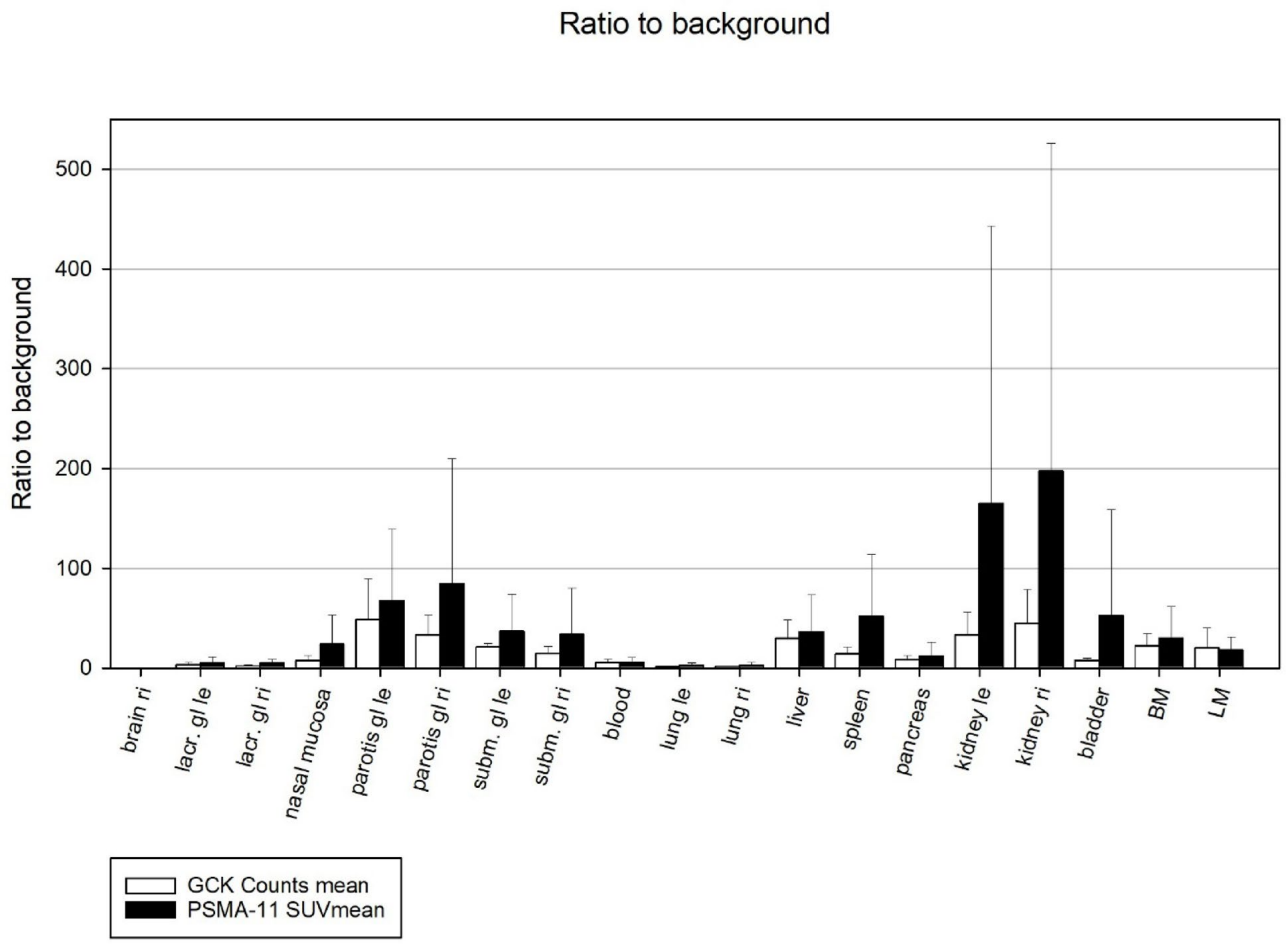
An overview of normal organ biodistribution and malignant lesion uptake, i.e. bone metastases (BM) and lymph node metastases (LM), with respect to background (pelvic fat tissue)

**Fig. 2:**
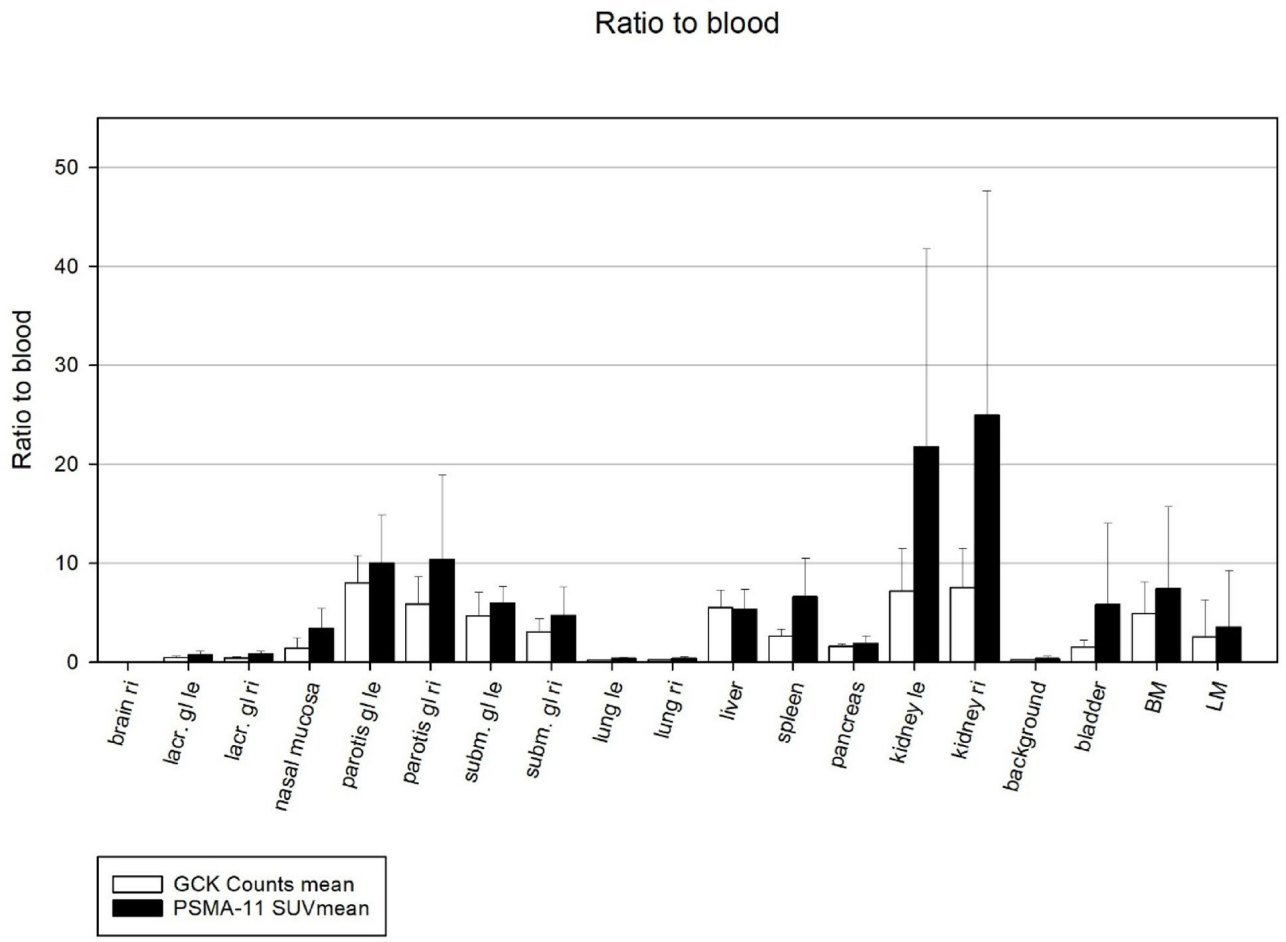
An overview of normal organ biodistribution and malignant lesion uptake, i.e. bone metastases (BM) and lymph node metastases (LM), with respect to blood pool in the descending aorta)

Compared to [^99m^Tc]Tc-PSMA-GCK01, [^68^Ga]PSMA-11 displayed a trend toward higher uptake in normal organs. This was reflected by target-to-background ratios using pelvic fat tissue and blood pool in the descending aorta as reference regions. TBR and TBP ratios were calculated within each modality as the VOI mean uptake in the target tissue (SUV_mean_ for PET; counts mean for SPECT) divided by the VOI mean uptake in the respective reference region (Figs. 1 and 2). Both tracers displayed higher uptake in the liver, kidneys and salivary glands compared with other normal organs. Using blood pool as reference, malignant lesions tended to exhibit more [^68^Ga]PSMA-11 uptake, whereas lymph node metastases showed higher lesion-to-background contrast (pelvic fat) with [^99m^Tc]Tc-PSMA-GCK01; these trends did not reach statistical significance for lymph node lesions (*p* > 0.05). In contrast, bone metastases demonstrated significantly higher target-to-blood pool ratios (VOImax) with [^68^Ga]PSMA-11 (*P* = 0.0001; Table 2).

### Assessment of biodistribution in phenotyping-critical organs

**Fig. 3:**
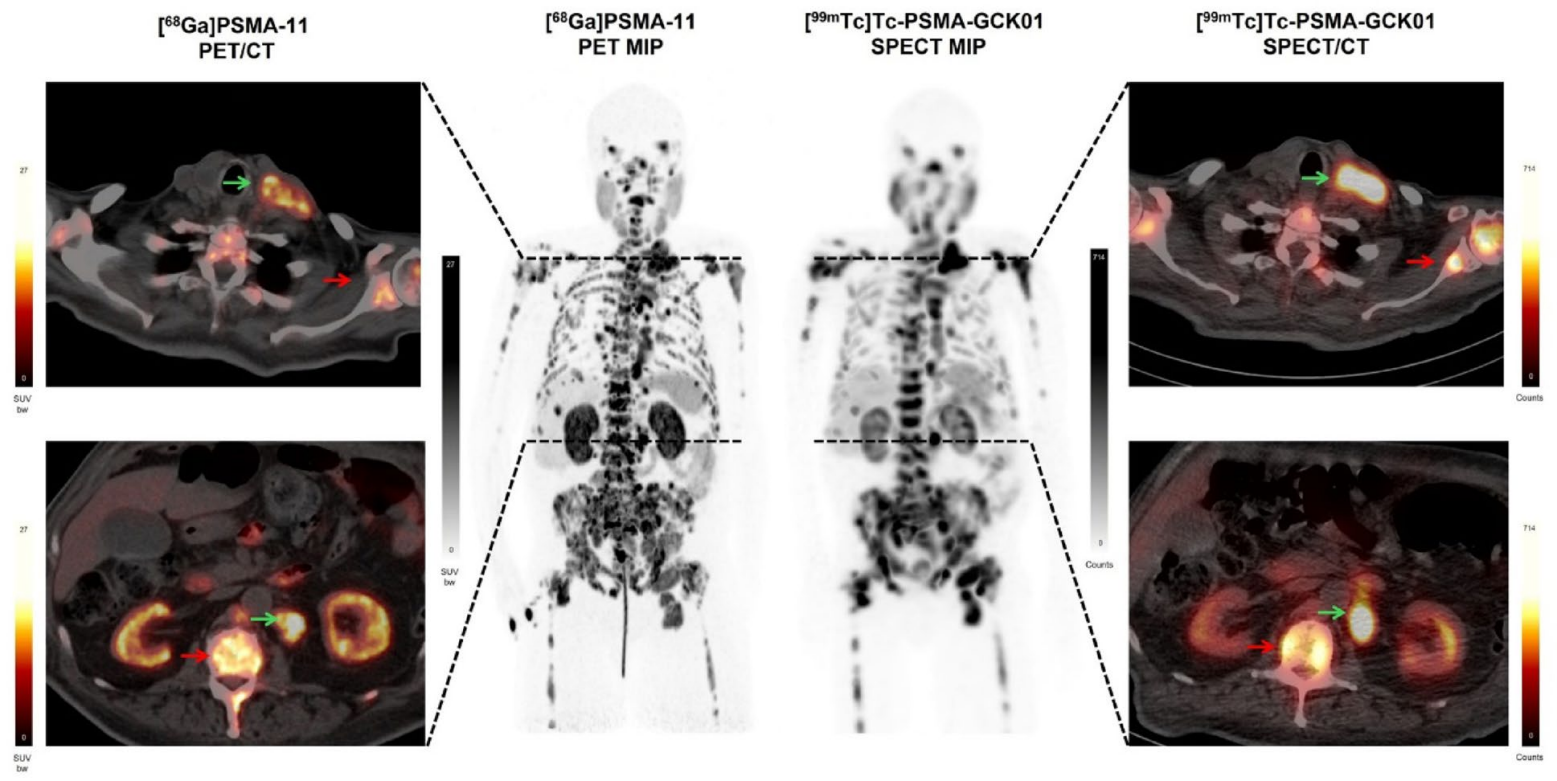
A 67-year-old patient with disseminated bone metastases in advanced stage of prostate carcinoma underwent both PSMA PET/CT and SPECT/CT scans for determination of phenotyping prior to RLT. Red arrows indicate bone metastases; green arrows indicate lymph node metastases. Interestingly, the high tracer uptake is also observed in the Virchow lymph nodes

**Fig. 4:**
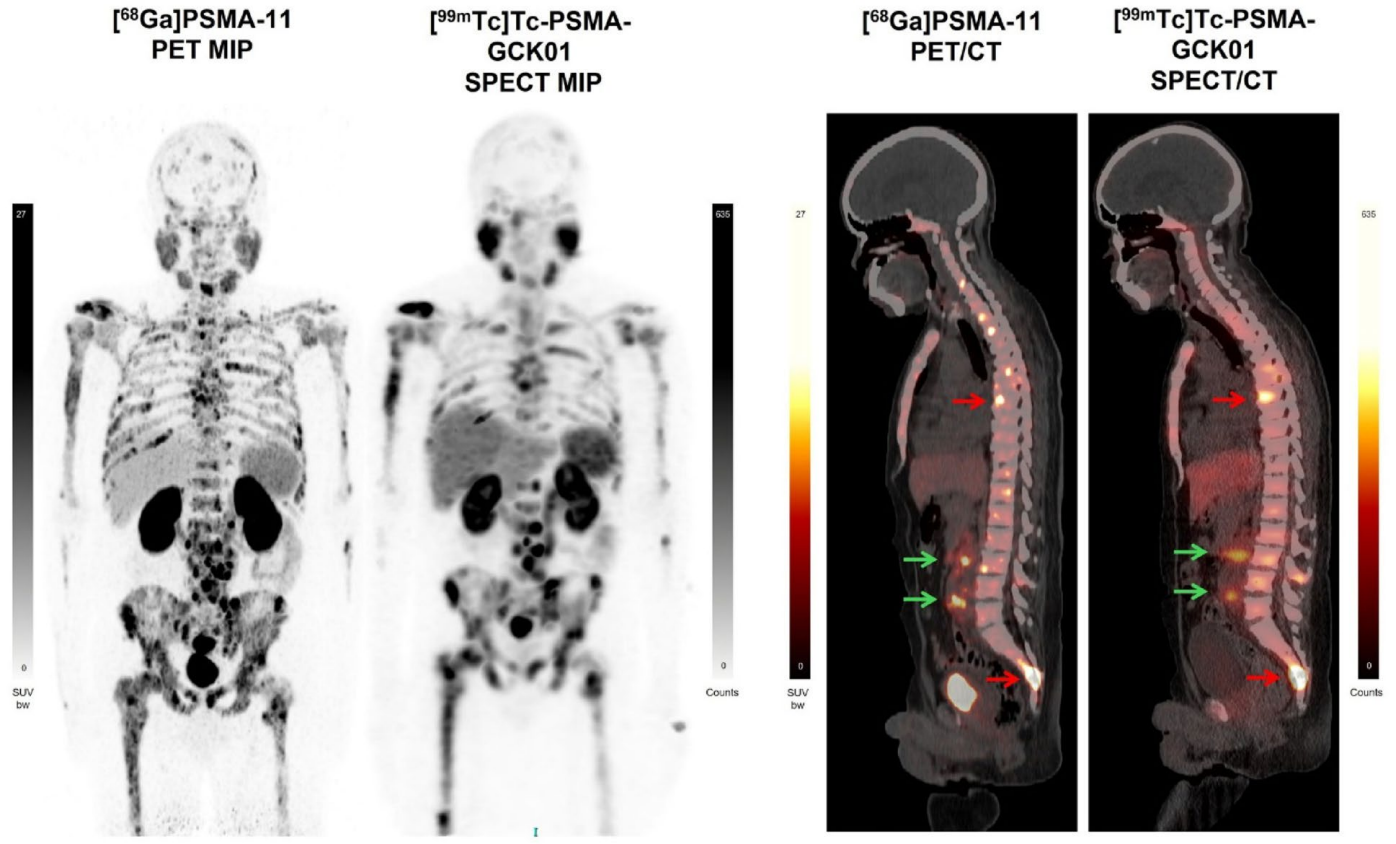
A 61-year-old patient with disseminated bone metastases in advanced stage of prostate carcinoma underwent both PSMA PET/CT and SPECT/CT scans for determination of phenotyping prior to RLT. Red arrows indicate bone metastases; green arrows indicate lymph node metastases

**Fig. 5:**
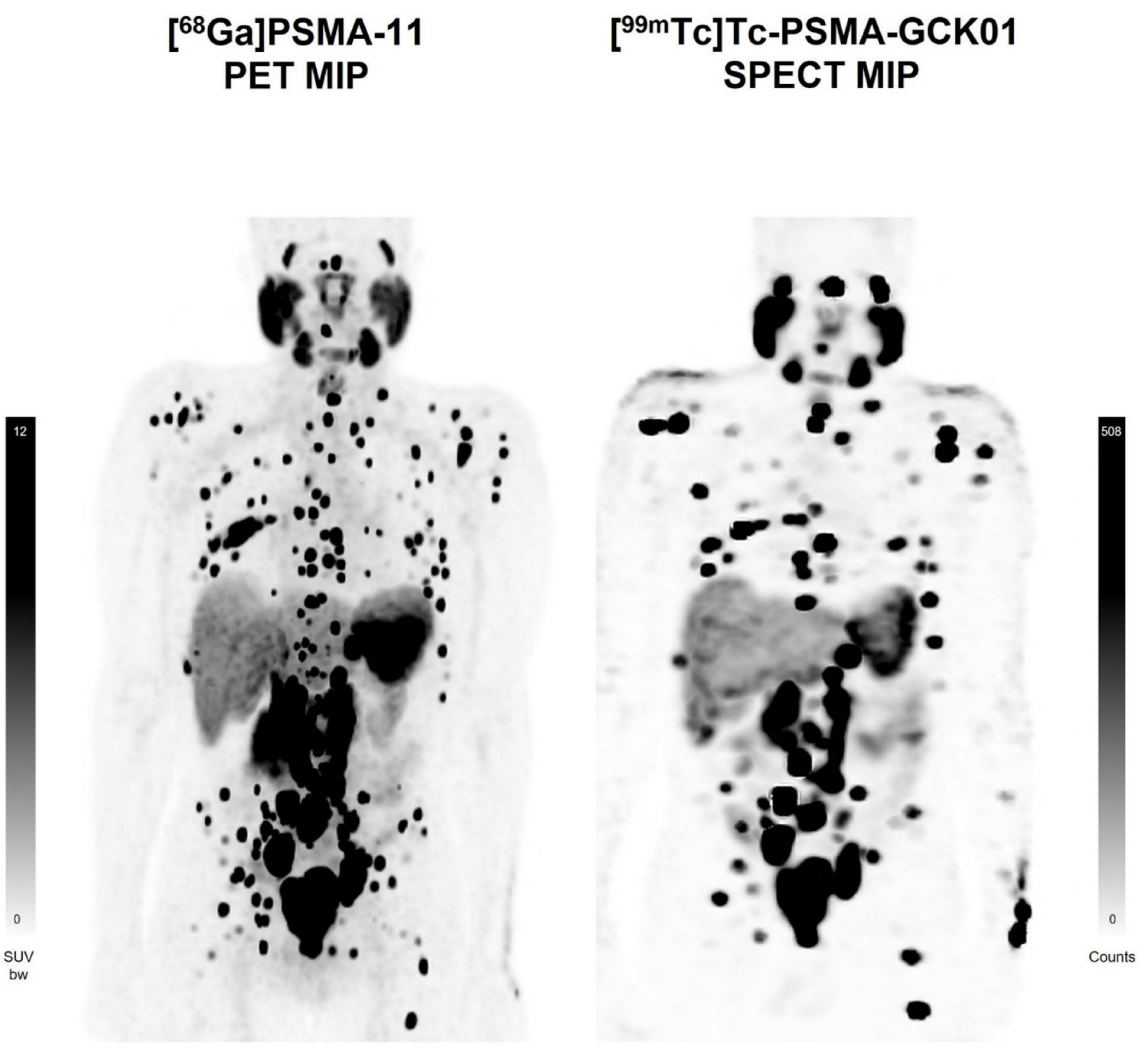
Metastases < 1 cm in diameter are often not delineable at SPECT resolution and metastases in the 1–2 cm diameter range are compromised e.g. by partial-volume effects, thus falsely underestimating tumor uptake of PSMA-ligand. Nevertheless, metastases > 2 cm demonstrate equivalent tumor-to-tissue contrast ratios (i.e. tumor vs. liver, spleen or salivary glands) with [^99m^Tc]Tc-PSMA-GCK01 SPECT compared with [^68^Ga]PSMA-11 PET. If only tumor lesions > 2 cm are considered, [^99m^Tc]Tc-PSMA-GCK01 SPECT stratifies the identical patients toward or against PSMA-RLT as [^68^Ga]PSMA-11 PET

Among normal organs, tracer uptake in the liver, kidneys, bone marrow and salivary glands is known to be critical for phenotyping and decision-making prior to RLT [11–13]. Thus, we assessed the tracer uptake for the aforementioned organs in a comparative manner based on the normalized ratio of Counts_mean_ versus SUV_mean_ and Counts_max_ versus SUV_max_ (Table 2). Renal uptake ratios were significantly higher with [^68^Ga]PSMA-11 than with [^99m^Tc]Tc-PSMA-GCK01. In the salivary glands and liver, uptake did not differ significantly between the tracers, although [^99m^Tc]Tc-PSMA-GCK01 tended to show lower uptake. Visual assessment also demonstrated intraindividual comparability of [^99m^Tc]Tc-PSMA-GCK01 and [^68^Ga]PSMA-11 *(*Figs. 3, 4 and 5*)*.

**Table 2:** Comparison of tracer biodistribution in decisive organs for RLT, which was quantified based on normalized background thresholds with pelvic fat tissue and blood pool in the descending aorta

Parameter	[^99m^Tc]Tc-PSMA-GCK01, median (min-max)	[^68^Ga]PSMA-11, median (min-max)	*P*
**Target/Background (VOImean)**			
Liver	24.5 (11–64)	26.4 (5.4–105)	0.76
Kidney	29.4 (6.8–100)	52.7 (11.8–854)	**0.002**
Bone Metastases	18 (7–46.4)	16.4 (1–107)	0.26
Submandibular gland	16.6 (9–29.6)	18.3 (5.1–126)	0.5
**Target/Background (VOImax)**			
Liver	17.7 (6.1–49.2)	15.1 (1.8–27.3)	0.56
Kidney	28.3 (1.3–89.5)	44.8 (6–163.6)	**0.027**
Bone Metastase	32.3 (1–117.3)	49.8 (12.7–113.4)	0.093
Gl. submandibularis	32.2 (1.4–84.2)	33.7 (6.4–68.1)	0.79
**Target/Blood pool (VOImean)**			
Liver	5.4 (2.3–7.5)	4.6 (2.9–8.4)	1.0
Kidney	7.3 (0.8–14.1)	18.1 (1.5–68.3)	**0.001**
Bone Metastases	4 (0.7–12.2)	5 (1–28.6)	0.64
Gl. submandibularis	3.8 (0.7–8.9)	5.1 (2.6–10.1)	0.07
**Target/Blood pool (VOImax)**			
Liver	4.6 (2.7–13.2)	4 (2.2–5.5)	0.15
Kidney	9.4 (2.3–12.8)	13.2 (5.4–32.9)	**0.002**
Bone Metastases	10.3 (1.5–29.7)	18.6 (5.1–33.2)	**0.0001**
Gl. submandibularis	8.2 (3–32.4)	8.6 (4.5–14.7)	0.68

Background VOI = pelvic fat tissue. Blood pool VOI = descending aorta. VOImean/VOImax denote ratios calculated using the mean or maximum uptake within the target VOI (within each modality). P values from paired statistical testing. Bold P values indicate statistical significance (*P* < 0.05)

## Discussion

The approval by regulatory bodies and implementation of PSMA theranostics employing [^68^Ga]PSMA-11 PSMA-targeting RLT in regular clinical care have shifted the standard of prostate cancer management. Accordingly, patients in remote or resource-limited regions may encounter logistical factors that may limit access to PSMA-targeting theranostics. Steadily increasing demand for phenotyping and therapy response monitoring of PSMA-RLT worldwide may benefit from novel approaches to expand access. The novel PSMA theranostics platform [^99m^Tc]Tc/[^188^Re]Re-PSMA-GCK01 described by Cardinale et al. may support phenotyping and enables generator-based ^188^Re-labelling for therapy purposes [8, 14].

This monocentric, prospective study with 10 patients with metastatic castration-resistant prostate cancer mCRPC and advanced multiorgan metastatic disease aimed to investigate the potential of [^99m^Tc]Tc-PSMA-GCK01 for PSMA phenotyping by evaluating biodistribution and tumor uptake in a head-to-head comparison with the current benchmark tracer [^68^Ga]PSMA-11. All patients were enrolled in this study consecutively by random choice following informed consent. Despite this approach, we cannot exclude a certain degree of selection bias due to small cohort size. Given the ethical issues with intraindividual comparison with different tracers, a larger patient cohort was neither desired nor feasible. To address the limited direct comparability of tracer uptake between PET and SPECT scan systems, we utilized normalized semi-quantitative metrics, i.e. TBR and TBP. These ratios are highly reproducible and effectively mitigate cross-modality calibration differences. However, the qualitative, visual assessment remains as the mainstay of clinical decision-making process for pre-therapeutic phenotyping prior to RLT in regular clinical care.

Visual assessment suggested qualitatively comparable lesion detection between [^99m^Tc]Tc-PSMA-GCK01 and [^68^Ga]PSMA-11 in this high–disease burden cohort. The statistical comparison of tracer uptake in normal organs revealed no statistically significant difference for most tissues, although [^99m^Tc]Tc-PSMA-GCK01 tended to display lower uptake in several organs (Fig. 1). In organs relevant to phenotyping and RLT planning (liver, salivary and lacrimal glands and kidneys), uptake ratios were broadly comparable with significantly lower renal uptake for [^99m^Tc]Tc-PSMA-GCK01 compared with [^68^Ga]PSMA-11 (Table 2).

These findings may be relevant for PSMA phenotyping in which liver activity is used as a reference (VISION trial) [1]. Given the comparable liver uptake between tracers (Table 2), application of liver-based thresholds used with [^68^Ga]PSMA-11 is unlikely to be confounded when using [^99m^Tc]Tc-PSMA-GCK01, although confirmation in larger cohorts is warranted. The significantly lower renal uptake observed for [^99m^Tc]Tc-PSMA-GCK01 (Table 2) may be relevant for treatment planning and could translate into different organ dosimetry for the therapeutic [^188^Re]Re-labelled analogue; however, clinical toxicity outcomes (including xerostomia) and tolerance of repeated RLT cycles were not assessed in this imaging study and require dedicated dosimetry and safety evaluation. Quantitative lesion uptake ratios were broadly comparable overall, with lesion-type–specific differences observed (Table 2).

The statistical comparability of liver uptake between the tracers, with nominally lower liver uptake for [^99m^Tc]Tc-PSMA-GCK01 compared with [^68^Ga]PSMA-11, supports the feasibility of applying liver-referenced phenotyping concepts with [^99m^Tc]Tc-PSMA-GCK01. We regard this as a key finding, as physiologic liver uptake varies across PSMA ligands (including fluorinated tracers and other ^99m^Tc-labelled PSMA tracers), which can complicate direct transfer of [^68^Ga]PSMA-11–based phenotyping criteria [15, 16]. Such variability has motivated efforts to define alternative thresholds for RLT eligibility, raising concerns regarding clinical comparability and reimbursement frameworks. For instance, given its hepatobiliary excretion pathway, liver is a less suitable reference organ for phenotyping with [^18^F]PSMA-1007. In addition, other potential reference organs, such as spleen and salivary glands, display relatively high variability of tracer uptake compared to [^68^Ga]PSMA-11, as Seifert et al. reported that using spleen as the reference organ would exclude up to 42% of patients from RLT [15, 16]. Higher liver uptake has also been reported for [^18^F]F-JK-PSMA-7, which could affect liver-referenced phenotyping [17]. In contrast, [^18^F]F-DCFPyl has been reported to display biodistribution similar to [^68^Ga]PSMA-11 including liver uptake, supporting its use for phenotyping [18]. Thus, using the salivary glands as reference organ for phenotyping may be misleading, as the uptake in salivary glands tend to vary substantially even with [^68^Ga]PSMA-11 [19, 20]. Nevertheless, [^99m^Tc]Tc-PSMA-GCK01 showed comparable uptake with PSMA-11 in the salivary glands.

Despite certain limitations, ^99m^Tc-labelled PSMA ligands, i.e. [^99m^Tc]Tc-MIP-1404, [^99m^Tc]Tc-PSMA-I&S, [^99m^Tc]Tc-PSMA-T4, [^99m^Tc]Tc-PSMA-11, [^99m^Tc]Tc-mas3-ynal-k(Sub-KuE), and [^99m^Tc]Tc-HYNIC-PSMA, have demonstrated promising results for primary staging and recurrence work-up in regular clinical care, including in resource-limited settings [7, 21–23]. Various studies have suggested a number of PSA levels as cut-off to ensure a consistently high diagnostic performance, as this PSA cut-off appeared to vary between 0.5 and 3.0 ng/ml [24]. Hence, setting a higher PSA cut-off value of > 1.0 ng/ml may facilitate the integration of PSMA SPECT ligands into regular clinical care due to increased diagnostic efficacy [25].

The prospective study by Ora et al. reported lower diagnostic performance of PSMA SPECT ligand for subcentrimetric lymph node metastases, whereas detection rate for other lesions was comparable to [^68^Ga]PSMA-11 [26]. Furthermore, PSMA SPECT ligands could be employed for therapy response monitoring, particularly in intermediate- and high-risk prostate cancer as well [27–29].

Given the comparability of [^99m^Tc]Tc-PSMA-GCK01 with [^68^Ga]PSMA-11 and the aforementioned data with other PSMA SPECT ligands, [^99m^Tc]Tc-PSMA-GCK01 may be suitable for additional components of PSMA theranostics beyond phenotyping; this requires validation in further studies. Currently, a dose-escalating-study with the use of [^188^Re]Tc-PSMA-GCK01 in mCRPC patients (RHINO-Trial) is underway to evaluate its safety profile and to support development of an RLT protocol for clinical use.

## Conclusion

[^99m^Tc]Tc-PSMA-GCK01 seems to be a viable, cost-effective complementary imaging modality for the current method of choice, [^68^Ga]PSMA-11, particularly for patients with late-stage disease for PSMA-phenotyping prior to PSMA-ligand therapies or interim staging. In addition, its accessibility makes it ideal for resource-limited settings in the landscape of Gamma camera-dominant geographical regions. Clinical safety and dosimetry are currently being validated in the RHINO phase 1/2a trial registered with the South African Clinical Trials Registry (*DOH-27-112024-4693*).

## Supplementary Information

Below is the link to the electronic supplementary material.

Supplementary Material 1.

## Data Availability

The data used and/or analyzed during the current study are available from the corresponding author upon reasonable request.
