# Supplementary material for: Evaluation of the biodistribution of [99mTc]Tc-PSMA-GCK01 SPECT/CT in a prospective, intraindividual comparison with [68Ga]PSMA-11 PET/CT for phenotyping in mCRPC prior to PSMA-targeted radioligand therapy
Source: EJNMMI Res. 2026 Aug 18;16:130. doi: 10.1186/s13550-026-01501-0 (PMC13486452; doi:10.1186/s13550-026-01501-0)
Supplement: Supplementary file 1 [file 13550_2026_1501_MOESM1_ESM.docx]

**Supplementary Table 1:** Acquisition Protocol for PET/CT and SPECT/CT imaging modalities.

| Modality | Unenhanced PET/CT | SPECT/CT |
| --- | --- | --- |
| Scanner | Biograph mCT 40 Siemens | Symbia Siemens doble-headed gamma camera |
| CT reference (mAs) | 40 | 30 |
| CT peak kilovoltage (kV) | 120 | 130 |
| CT slice thickness (mm) | 1,5 | 3 |
| CT slice increment (mm) | 1 | 1 |
| Reconstruction | OSEM algorithm | OSEM algorithm |
| Iterations | 4 | 8 |
| Subsets | 8 | 4 |
| Matrix | 200 x 200 | 128 × 128 |
| Corrections | Gaussian FWHM 5.0 mm | Gaussian FWHM 2.0 mm |
